# Oral leukoplakia, the ongoing discussion on definition and terminology

**DOI:** 10.4317/medoral.21007

**Published:** 2015-10-09

**Authors:** Isaäc van der Waal

**Affiliations:** 1 VU University Medical Center (VUmc)/Academic Centre for Dentistry Amsterdam (ACTA), Department of Oral and Maxillofacial Surgery and Oral Pathology, P.O. Box 7057, 1007 MB Amsterdam, The Netherlands

## Abstract

In the past decades several definitions of oral leukoplakia have been proposed, the last one, being authorized by the World Health Organization (WHO), dating from 2005. In the present treatise an adjustment of that definition and the 1978 WHO definition is suggested, being : “A predominantly white patch or plaque that cannot be characterized clinically or pathologically as any other disorder; oral leukoplakia carries an increased risk of cancer development either in or close to the area of the leukoplakia or elsewhere in the oral cavity or the head-and-neck region”. Furthermore, the use of strict diagnostic criteria is recommended for predominantly white lesions for which a causative factor has been identified, e.g. smokers’ lesion, frictional lesion and dental restoration associated lesion. A final diagnosis of such leukoplakic lesions can only be made in retrospect after successful elimination of the causative factor within a somewhat arbitrarily chosen period of 4-8 weeks. It seems questionable to exclude “frictional keratosis” and “alveolar ridge keratosis” from the category of leukoplakia as has been suggested in the literature. Finally, brief attention has been paid to some histopathological issues that may cause confusion in establishing a final diagnosis of leukoplakia.

** Key words:**Oral leukoplakia, potentially malignant oral disorders, definition.

## Introduction

1. The definition and terminology of oral leukoplakia and leukoplakialike (“leukoplakic”) lesions and disorders of the oral mucosa is the subject of discussion in the literature for many decades. This discussion is mainly focused on clinical aspects, but is partly related to some histopathological aspects as well. In this treatise the various definitions of oral leukoplakia will be discussed, resulting in a suggestion for a slight adjustment of the 2005 WHO definition. Furthermore, some leukoplakic lesions will be discussed that may cause some confusion as whether or not to exclude them from the category of leukoplakia; examples are “alveolar ridge keratosis” and “frictional keratosis”.

## Definition

2.1 The various WHO definitions and suggestion for an adjusted definition

In 1978, oral leukoplakia has been defined by the World Health Organization (WHO) as: ‘A white patch or plaque that cannot be characterized clinically or pathologically as any other disease’ (1). In an explanatory note it has been explicitly stated that the term leukoplakia is unrelated to the absence or presence of epithelial dysplasia. In a monograph by the WHO, published in 1997, the phrase: ‘any other definable disease’ was replaced by ‘any other definable lesion’ (2). No justification has been provided for this change.

**Figure 1:**
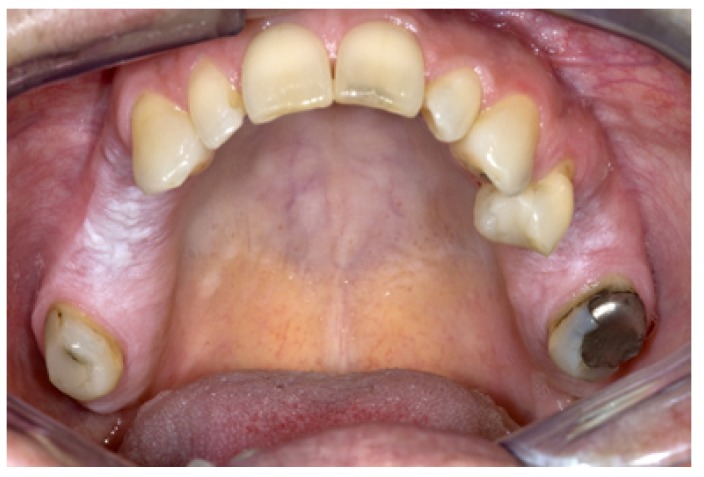
Leukoplakia (or "benign alveolar ridge keratosis"?) in both sides of the maxilla in a patient who never smoked and has not been wearing a partial denture.

In 2002, it has been recommended to make a distinction between a provisional clinical diagnosis of oral leukoplakia and a definitive one ( Table 1) (3). A provisional diagnosis was made when a lesion at the initial clinical examination could not be clearly diagnosed as either leukoplakia or any other disease. In case of a provisional clinical diagnosis, Certainty factor 1 was assigned ( Table 2). A definitive clinical diagnosis of leukoplakia was made after unsuccessful elimination of suspected etiological factors or in the absence of such factors, assigning Certainty factor 2. Certainty factor 3 was assigned when histopathological examination of an incisional biopsy did not show the presence of any other diseases. In case of an excisional biopsy or surgical excision, performed after an incisional biopsy, Certainty factor 4 was assigned based on histopathological examination of the surgical specimen (Fig. 1). It goes without saying that in epidemiological studies a Certainty factor 1, based on a single oral examination, is acceptable, while in scientific studies, e.g. comparing different treatment results, Certainty factor 4 will be required, if feasible. Apparently, the recommendation to use a Certainty factor has not been widely accepted in the recent literature (4), although the use of such factor is common practice in cancer registries.

**Table 1:**
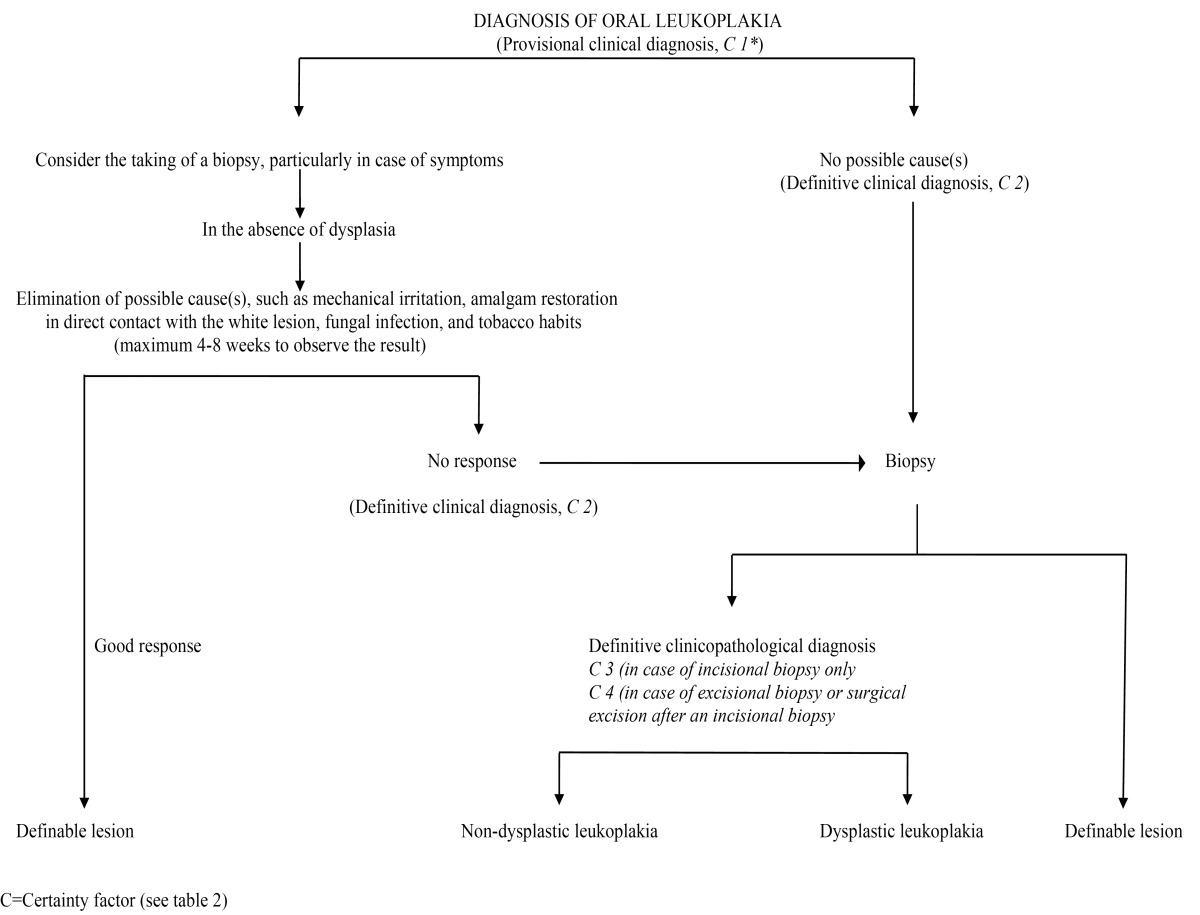
Diagnosis of oral leukoplakia.

**Table 2:**
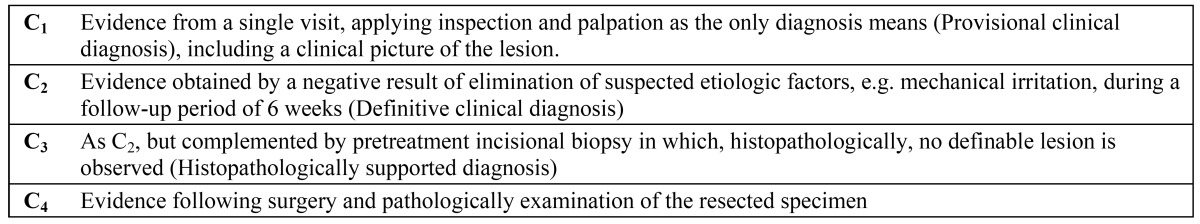
Certainty (C)-factor of a diagnosis of oral leukoplakia.3.

In 2005, the definition of oral leukoplakia has been changed at a WHO supported meeting into: ‘A white plaque of questionable risk having excluded (other) known diseases or disorders that carry no increased risk for cancer’ (5). During the latter meeting it has deliberately been decided to consider leukoplakia a potentially malignant- pre malignant and pre cancerous are equivalent adjectives- disease and not a lesion since it is well known that cancer development not always occurs in or close to the leukoplakia but may also occur at other sites in the oral cavity or the head-and-neck region.

Although various international meetings on the subject of oral leukoplakia have been held between 1978 (WHO) and 2005 (WHO) no substantial changes in the definition of leukoplakia have resulted from these meetings.

In the sixties of the past century a minimum size of 5 mm was required before being allowed to use the term leukoplakia (6). There seems no strong reason to reintroduce a minimum size, since cancer devopment may also take place in very small leukoplakias. Another part of previous definitions of leukoplakia has been the requirement of a non-removable nature of the white lesion, apparently mainly meant to separate pseudo membranous candidiasis from leukoplakia. The adjectives “non-removable” or “non-scrap able” seem, indeed, to have some practical value, but there is no strong reason to include these in the definition.

The advantage of the 2005 WHO definition above the one from 1978 is its statement about the behaviour of oral leukoplakia (“questionable risk”). Unfortunately, in both WHO definitions (1978 and 2005) the diagnosis of leukoplakia is one by exclusion (of other “known diseases or disorders”). A list of these “known diseases” is depicted in  Table 3. Some of these diseases will be briefly commented upon (see ad 3). Many, if not most, of the listed entities may be easy to distinguish from leukoplakia by an experienced clinician, either based on the history or based on the clinical appearance, but this may not be the case for the less experienced clinician, being either a dentist, an oral and maxillofacial surgeon, an otolaryngologist or a dermatologist. Furthermore, it does not seem realistic to expect family doctors to be knowledgeable in this field, since probably in most parts of the world little attention is paid to oral diseases during the medical curriculum. One may even discuss at what level dentists-general practitioners should be educated in the diagnosis and management of the numerous lesions and disorders of the oral mucosa .

**Table 3:**
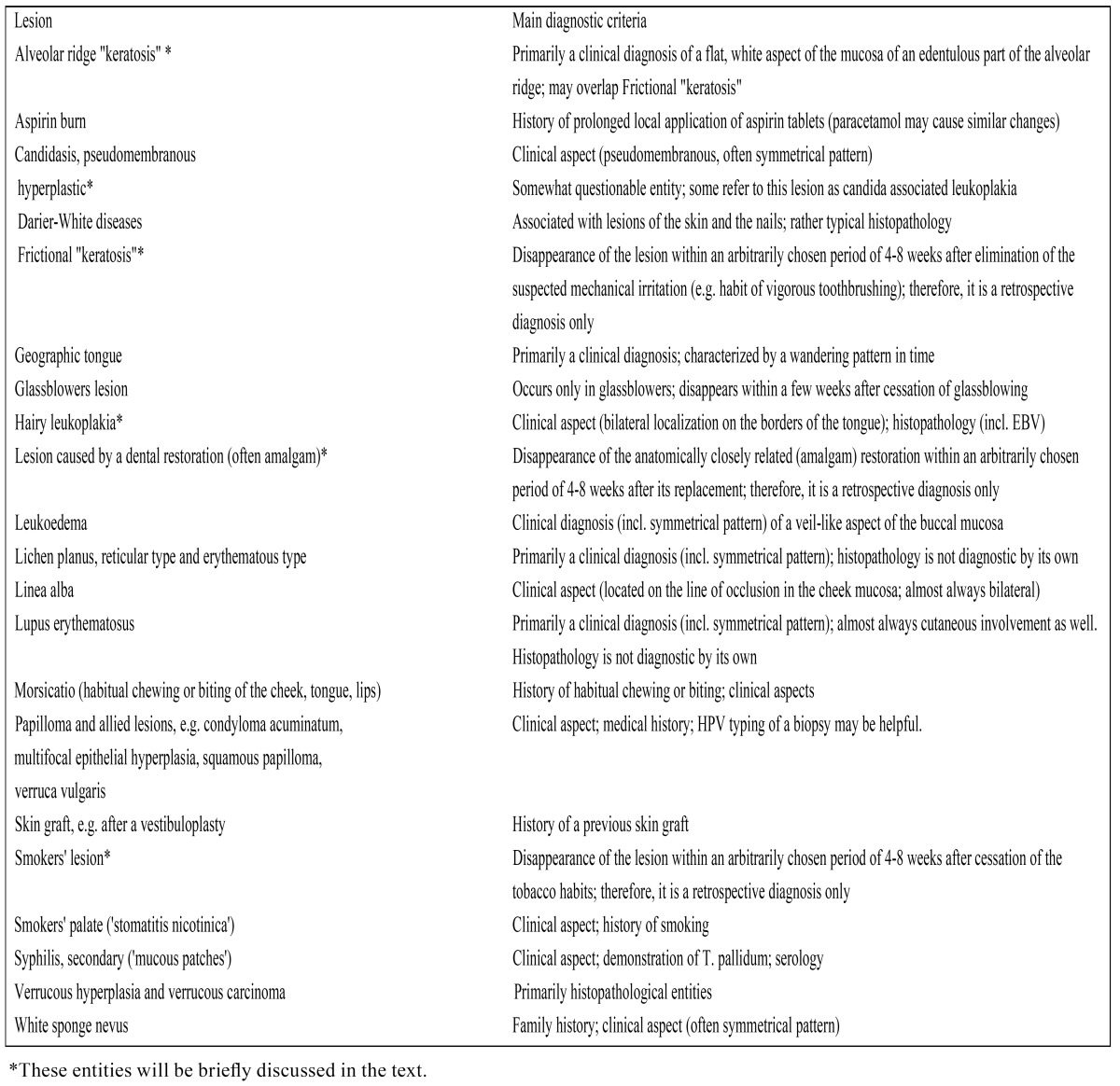
Definable white diseases and disorders that may have a leukoplakic appearance.

A combination of the 1978 and the 2005 WHO definitions of oral leukoplakia may result in the following text: “A predominantly white patch or plaque that cannot be characterized clinically or pathologically as any other disorder; oral leukoplakia carries an increased risk of cancer development either in or close to the area of leukoplakia or elsewhere in the oral cavity or the head-and-neck region”.

2.2 What is a significant increased risk of cancer development?

In defining a potentially malignant disorder it is usually stated that there is “significant increased risk” of cancer development, without specifying “significant”. When the incidence- number of new patients per year- of oral cancer is set at a low 2:100,000 population and the annual malignant transformation rate of leukoplakia at 2:100 (irrespective of the discussion whether or not treatment of leukoplakia reduces the risk of malignant transformation) there is a thousand fold risk in leukoplakia patients to develop cancer in comparison with patients not having leucoplakia. Probably, a thousand fold increased risk is perceived, particularly by patients, as being significant.

## Discussion on some “other known diseases and disorders” that may have a leukoplakic appearance

-3.1 Alveolar ridge “keratosis”

A few papers have been devoted to “alveolar ridge keratosis” (7,8). Apparently, the supposed cause of the lesion is chronic frictional (masticatory) trauma to the maxillary and mandibular alveolar ridges. Histopathologically, almost of all these lesions show hyperkeratosis without epithelial dysplasia. The suggestion has been made in both previous papers to remove this lesion from oral leukoplakia, mainly based on the assumption that malignant transformation is extremely rare (Fig. 1). There are not many follow-up studies that focused on leukoplakia of the alveolar ridges only. Therefore, there is some reluctance to accept the suggestion to remove this lesion from leukoplakia.

In one paper on this subject it was noted that alveolar ridge keratosis resembles chronic lichen simplex of the skin, apparently being caused by chronic frictional injury (8). Therefore, the authors suggested the somewhat confusing term “oral lichen simplex chronicus” as a synonym.

There may be some overlap with the reported frictional keratosis of the facial (buccal) attached gingiva to be discussed in 3.3

-3.2 Candidiasis, hyperplastic type

There is some room for discussion, both clinically and histopathologically, about the diagnosis of hyper plastic candidiasis versus Candida-associated leukoplakia, particularly if located at the commissures of the lips, the hard palate and the dorsal surface of the tongue. If such lesions disappear after anti fungal treatment within an arbritarily chosen period of 4-8 weeks there is no justification to call such lesions leukoplakias any longer. However, in case of persistence, it seems safe practice to consider a diagnosis of Candida-associated leukoplakia.

-3.3 Frictional “keratosis” 

**Figure 2:**
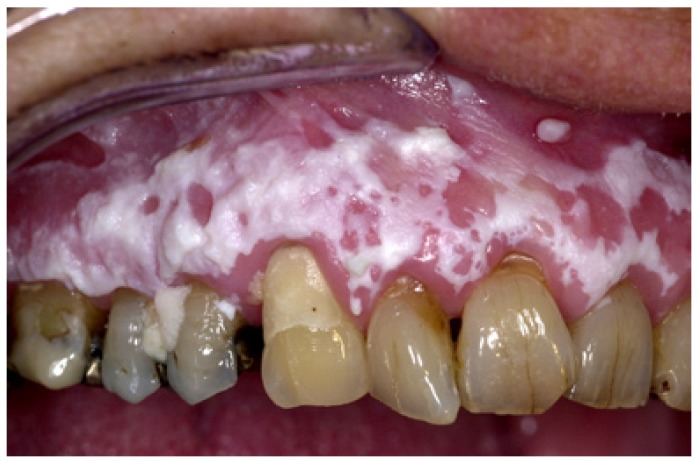
Leukoplakia (or "frictional keratosis"?) in a 57-year-old woman.

Another possible reversible white lesion is the frictional lesion caused by mechanical irritation, e.g. vigorously brushing of the teeth (Fig. 2). This lesion is sometimes referred to as “frictional keratosis”. The term “lesion” is preferred because “keratosis” is actually a histopathological term. There may be some overlap with the previously discussed alveolar ridge keratosis (see ad 3.1). The suggestion to remove this lesion- particularly when located on the buccal attached gingiva- from the category of leukoplakia seems rather questionable (9). A final diagnosis of frictional lesion can only be applied to cases where the lesion has disappeared after elimination of the possible mechanical cause- provided that there are no symptoms that would require to immediately biopsy the lesion- , within a somewhat arbitrarily chosen period of no more than 4-8 weeks. In other words, a definitive diagnosis of frictional lesion can only be made in retrospect.

-3.4 Hairy leukoplakia

The term hairy leukoplakia is a misnomer- but well accepted in the literature- for various reasons, being 1) it is a well described entity, particularly by the immunohistochemical demonstration of EBV DNA in the koilocytic epithelial cells of a biopsy specimen, 2) it is not a potentially malignant disorder, and 3) the clinical aspect is not always “hairy”. Admittedly, it is difficult to come up with a better term (10).

-3.5 Restoration associated lesion

A somewhat similar approach as discussed above with regard to frictional lesions is valid in case of a provisional diagnosis of a “contact lesion”, supposedly caused by direct prolonged contact by large amalgam restorations, particularly in case of a buccal or lingual extension. A final diagnosis of amalgam associated lesion should only be applied when the lesion has disappeared after replacement or removal of the amalgam restoration- provided that there are no symptoms that would require to immediately biopsy the lesion- , within a somewhat arbitrarily chosen period of no more than 4-8 weeks. Therefore, a definitive diagnosis of amalgam associated lesion can only be made in retrospect.

-3.6 Smokers’ lesion versus tobacco associated leukoplakia

It is well known that leukoplakia in patients with tobacco habits might be reversible if patients give up their smoking habits (11). In the absence of symptoms, being a strong indication for an immediate biopsy in order to exclude the presence of severe epithelial dysplasia or even squamous cell carcinoma, the patient should be advised to give up the tobacco habit. If successful and if the white lesion regresses within a somewhat arbitrarily chosen period of no more than 4-8 weeks the provisional clinical diagnosis of such lesion should, in retrospect, be changed into “smokers’ lesion”. When the patient is not able or willing to give up the tobacco habit and in case of persistence of the leukoplakia, the term “tobacco-associated leukoplakia” can be applied, irrespective of the relevance of such designation.

## Clinical classification of leukoplakia with emphasis on (proliferative) verrucous leukoplakia.

4 1. Traditionally, two major clinical types of leukoplakia are recognized, being the homogeneous and the non-homogeneous type respectively. The significance of this classification is the assumption that there is a correlation between the clinical type and the risk of malignant transformation, the non-homogeneous type carrying a higher risk. In some studies there is such correlation while in other studies there is not.

**Figure 3:**
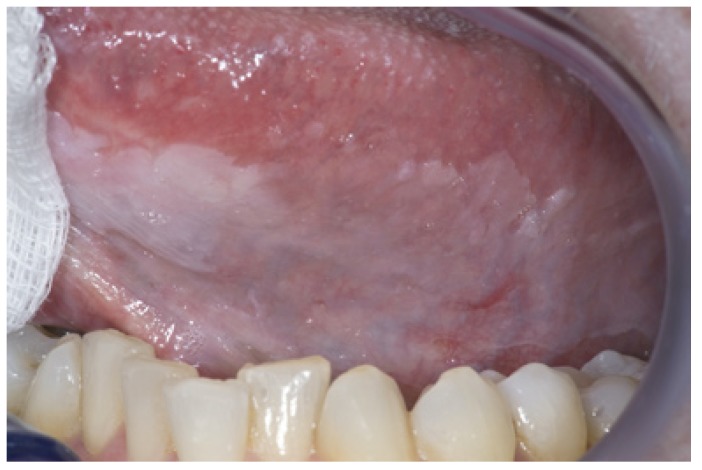
Homogeneous (flat and thin) leukoplakia in a 53-year-old man.

**Figure 4:**
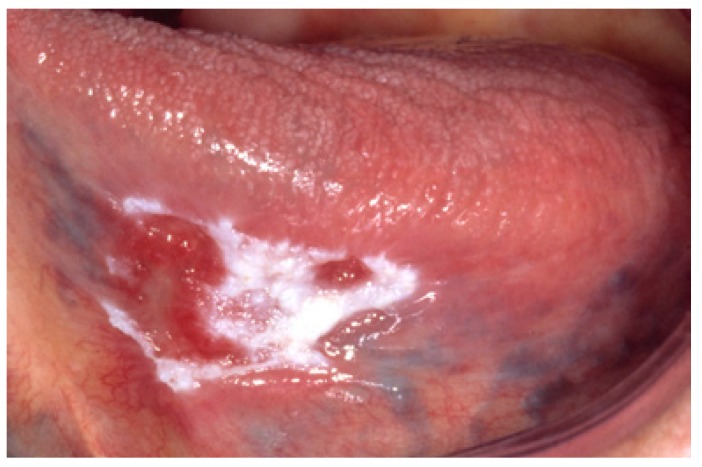
Non-homogeneous (white and red changes, also referred to as erythroleukoplakia) in an 88-year-old woman.

**Figure 5:**
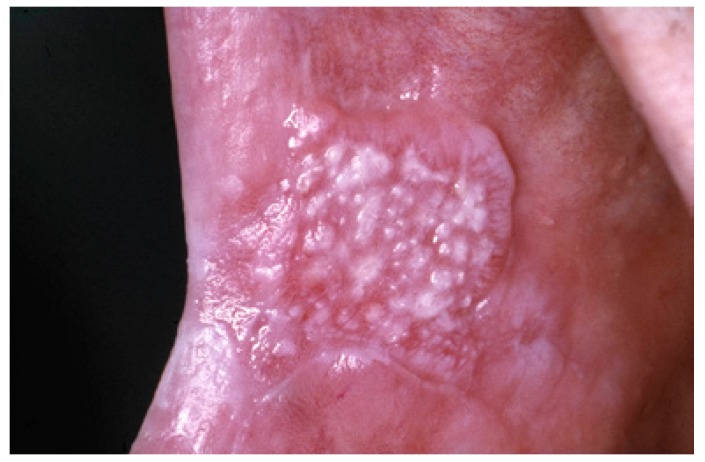
Non-homogeneous, nodular, leukoplakia in a 61-year-old man.

**Figure 6:**
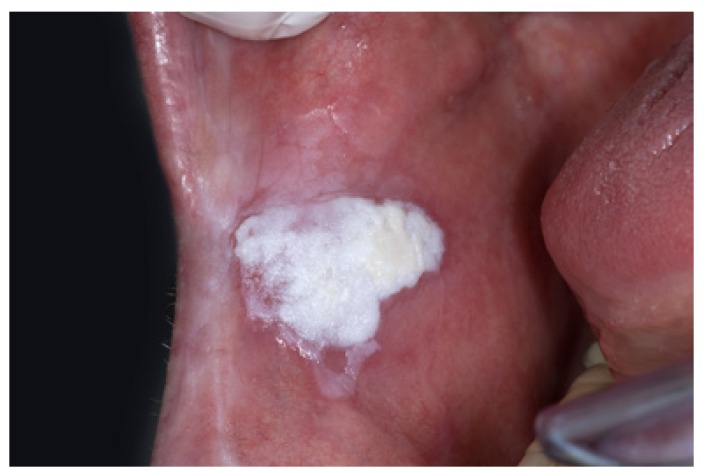
Non-homogeneous, verrucous leukoplakia. In spite of a homogeneous white appearance and a homogeneous verrucous texture, this lesion should not be called homogeneous leukoplakia

The homogeneous type is characterized by a thin, flat and homogeneous whitish appearance (Fig. 3). The non-homogeneous type is subdived in a variety of subtypes, such as speckled or erythematous (white and red changes), also referred to as erythroleukoplakia (Fig. 4), nodular (Fig. 5) and verrucous (Fig. 6). Particularly the verrucous type is probably quite often misdiagnosed by clinicians because of its homogeneous white appearance and its often homogeneous (verrucous) texture. There are actually no strict criteria how to make a distinction clinically between verrucous leukoplakia and verrucous carcinoma.

Another confusing type is the proliferative verrucous leukoplakia (PVL), as being introduced in the literature by Hansen *et al*. (12). In the original publication PVL has been characterized as a slow-growing, persistent, and irreversible lesion, resistant to all forms of therapy as recurrence is the rule. PVL may start as a simple keratosis at one end to invasive carcinoma at the other.In fact, a diagnosis of PVL can only be made in retrospect. In several of such cases the initial lesion may just be a solitary homogeneous or non-homogeneous leukoplakia (13). Unfortunately, in many scientific reports on PVL just multifocality and involvement of the gingiva seem to have been used as diagnostic criteria without paying attention to the history of the disease.

## Some histopathological areas of confusion

-5.1 The assessment of epithelial dysplasia

It is well recognized that the assessment of the presence and degree of epithelial dysplasia carries a substantial degree subjectivity, reflected in a distinct intra- and inter observer variation (14-16). Unfortunately, in spite of numerous attempts, as being suggested in the literature, there is no international consensus on this issue.

Probably some pathologists will deny a diagnosis of leukoplakia in the absence of epithelial dysplasia, what is not in accordance with the recommendations from the “dental” literature.

-5.2 Lichenoid dysplasia

In 1985 the supposedly distinct entity of lichenoid dysplasia has been introduced in the literature (17). The use of of this term is discouraged since it, erroneously, may suggest dysplastic changes occurring in oral lichen planus. Probably a number of the reported cases of malignant transformation of lichen planus are caused by forementioned confusing terminology, while cases of leukoplakia with a lichenoid appearance histopathologically, mainly consisting of a sub epithelial bandlike infiltrate, may have erroneously been reclassified as lichen planus.

-5.3 Verrucous hyperplasia versus verrucous carcinoma

Several papers have been published about the histopathological difference between verrucous hyperplasia and verrucous carcinoma, still leaving room for discussion (18,19). In daily practice it is difficult, if not impossible, to make this distinction in a reproducible way. Besides, one may question the clinical relevance of the distinction between these two entities since for both lesions (surgical) removal is recommended. A pitfall is that some pathologists may describe these epithelial changes as being benign, while the behavior of such lesions actually is unpredictable.

## Discussion and Conclusions

It is well appreciated that a number of aspects of the presently discussed definition and terminology may not be equally valid in all parts of the world. A classification of potentially malignant disorders has been proposed in 2011 from India (20). Apparently, this classification is not limited to leukoplakia, but also includes entities such as lichen planus, oral sub mucous fibrosis, nutritional deficiencies and some inherited cancer syndromes.

The recommendation is made to modify the present 2005 WHO definition of oral leukoplakia, amongst others by adding explicitly the requirement of histopathologic examination in order to obtain a definitive clinicopathological diagnosis. As a result, the following definition is proposed: “A predominantly white patch or plaque that cannot be characterized clinically or pathologically as any other disorder; oral leukoplakia carries an increased risk of cancer development either in the area of the leukoplakia or elsewhere in the oral cavity or the head-and-neck region”.

Furthermore, the use of strict diagnostic criteria is recommended for predominantly white lesions or diseases for which a possible causative factor has been identified, e.g. smokers’ lesion, frictional lesion and dental restoration associated lesion. An observation of 4-8 weeks after removal of the suggested cause seems a practical one and seems also safe practice, particularly in case of an asymptomatic leukoplakic disorder. In this respect one should realize that at the first visit of a patient with oral leukoplakia a squamous cell carcinoma may be present already and one would not run the risk of observing such event for a period of more than 4-8 weeks. Even such period is already a long one in case of a squamous cell carcinoma, a carcinoma in situ or severe epithelial dysplasia. However, it should be emphasized, that the presence of such changes is nearly always associated with symptoms. Therefore, in the presence of symptoms a biopsy is strongly recommended before elimination of possibly causative factors and observation of the result of such elimination.

As is true for almost all pathologies proper communication between clinicians and pathologists is important, particularly in the field of oral potentially malignant disorders. For instance, some pathologists will deny a diagnosis of leukoplakia in the absence of epithelial dysplasia. Also the use of the term “lichenoid dysplasia” may be the subject of confusion between pathologists and clinicians.
